# Combinatorial network of transcriptional regulation and microRNA regulation in human cancer

**DOI:** 10.1186/1752-0509-6-61

**Published:** 2012-06-12

**Authors:** Hui Yu, Kang Tu, Yi-Jie Wang, Jun-Zhe Mao, Lu Xie, Yuan-Yuan Li, Yi-Xue Li

**Affiliations:** 1 Key Lab of Systems Biology, Shanghai Institutes for Biological Sciences, Chinese Academy of Sciences, 320 Yueyang Road, Shanghai, 200031, People's Republic of China; 2 Shanghai Center for Bioinformation Technology, 100 Qinzhou Road, Shanghai, 200235, People's Republic of China; 3 School of Life Science and Biotechnology, Shanghai Jiaotong University, 800 Dongchuan Road, Shanghai, 200240, People's Republic of China; 4 Shanghai High School, 989 Baise Road, Shanghai, 200231, People's Republic of China

## Abstract

****Background**:**

Both transcriptional control and microRNA (miRNA) control are critical regulatory mechanisms for cells to direct their destinies. At present, the combinatorial regulatory network composed of transcriptional regulations and post-transcriptional regulations is often constructed through a forward engineering strategy that is based solely on searching of transcriptional factor binding sites or miRNA seed regions in the putative target sequences. If the reverse engineering strategy is integrated with the forward engineering strategy, a more accurate and more specific combinatorial regulatory network will be obtained.

****Results**:**

In this work, utilizing both sequence-matching information and parallel expression datasets of miRNAs and mRNAs, we integrated forward engineering with reverse engineering strategies and as a result built a hypothetical combinatorial gene regulatory network in human cancer. The credibility of the regulatory relationships in the network was validated by random permutation procedures and supported by authoritative experimental evidence-based databases. The global and local architecture properties of the combinatorial regulatory network were explored, and the most important tumor-regulating miRNAs and TFs were highlighted from a topological point of view.

****Conclusions**:**

By integrating the forward engineering and reverse engineering strategies, we manage to sketch a genome-scale combinatorial gene regulatory network in human cancer, which includes transcriptional regulations and miRNA regulations, allowing systematic study of cancer gene regulation. Our work establishes a pipeline that can be extended to reveal conditional combinatorial regulatory landscapes correlating to specific cellular contexts.

## **Background**

Transcriptional factors (TF) and microRNAs (miRNAs) are important regulation factors to determine the expression levels of mRNAs and miRNAs [1]. TFs activate or repress gene transcription by binding to specific sites (transcription factor binding sites, or TFBSs) in promoter regions, thus regulating gene expression at the transcription level; miRNAs inhibit mRNA translation by inducing mRNA degradation and/or blocking the translation machinery, thus negatively regulates gene expression at the post-transcriptional level. Given the facts that the transcription of both mRNA and miRNA is regulated by TFs, and that mRNA expression, including TF’s, could be modulated by miRNAs, the cellular transcriptome is believed to be determined by combinatorial regulatory network of at least two interconnected layers, where TFs work as master regulators in the transcriptional layer and miRNAs as fine tuners in the post-transcriptional layer [1]. It thus becomes critical to delineate and characterize the two-layered combinatorial regulatory networks, for the sake of understanding the regulatory mechanisms at a higher precision than what we can do with either layer alone.

Databases, such as TransFAC [2] on TF-to-mRNA regulation, TransmiR [3] on TF-to-miRNA regulation, and TarBase [4] on miRNA-to-mRNA regulation, provide experimentally validated regulation relationships between regulators and their targets. However, such data alone are too limited to enable large-scaled studies. Therefore, peers have resorted to a forward-prediction strategy to infer regulatory relationships between TFs or miRNAs and their putative targets based on the matching or complementary of motif or seed sequences [5,6]. In this way, they built the two-layered combinatorial regulatory networks, and investigated the global and local architectural properties [7,8]. It is imaginable that a high rate of false positive predictions is necessitated [9], and moreover, these forward works generate ‘reference networks’ that span across all spatiotemporal contexts – in concept all regulations that take place at different temporal points and different cells or tissues are combined unreasonably. That is, forward engineering cannot solve a conditional regulatory network that corresponds to a particular cellular context. The reverse engineering strategy therefore comes into use where the regulatory relationships between TFs or miRNAs and their putative targets (cause) are inferred from the observed expression correlations (consequence) (for a review see [10]).

Reverse engineering has been put into effect in inferring TF-controlled transcriptional regulation networks [11-13] as well as sifting miRNA potential targets [14,15]. However, we have rarely seen successful applications of reverse engineering in inferring combinatorial networks involving TFs and miRNAs, except for a few works where small-scaled combinatory circuits of miRNAs and TFs were mapped around some selected genes prioritized from the expression data [16-18]. The major obstacle in this direction, lack of simultaneously measured miRNA expression data and mRNA expression data, is being relieved as parallel miRNA expression and mRNA expression datasets are being continuously released to public [19], such as those for epithelial samples [20,21] or various tumor samples [22-24]. Having only been explored for confirming predicted miRNA targets [24,25] or extracting tumor-classifying molecular signatures [26], these parallel expression datasets have far more potential to be exploited.

Previously, we integrated forward predicted gene regulation relationships with miRNA-perturbed gene expression datasets (MPGE datasets) and as a result elucidated miRNA-centered primary and secondary regulatory cascades in human cancer by using nonparametric test and linear regression modeling [27]. Confined to the type of expression data - mRNA expression, the combinatorial regulatory networks mapped therein encompassed only the regulation of mRNA by TF and by miRNA (miRNA-to-mRNA, TF-to-mRNA), missing the regulation of miRNA by TF (TF-to-miRNA). This limitation is also existent in a contemporary study [28], which substitutes mRNA’s expression data for that of the embedded intragenic miRNA in order to identify miRNA-mediated feedback and feed-forward loops. We realize that studies on combinatorial gene regulatory network can be advanced significantly with the help of the aforementioned parallel miRNA expression and mRNA expression datasets. Due to our preceding work on human cancers [27], we are particularly interested in the NCI-60 data panel [23,29] which involves 60 cancerous cell lines originating from breast, central nervous system, colon, leukemia, melanoma, Non-Small Cell Lung, ovarian, prostate, and renal tissues.

In the present work, we demonstrated an efficient integration of the forward-predicted candidate regulatory relationships with the NCI60 panel of parallel miRNA and mRNA expression datasets, giving rise to a genome-scale combinatorial network of transcriptional regulations and miRNA regulations in human cancer. The resultant combinatorial regulatory network makes a scaffold for systematic study of cancer gene regulation, and the demonstrated working pipeline can be extended to reveal conditional combinatorial regulatory landscapes in other cellular contexts.

## **Materials and methods**

### **Parallel miRNA and mRNA expression datasets**

From the NCI-60 data source CellMiner (http://discover.nci.nih.gov/cellminer/), we downloaded the NCI-60 mRNA expression dataset assayed with the Affymetrix HG-U133 44 K probeset microarray [29] and the parallel miRNA expression dataset assayed with the OSU-CCC-hsa-miRNA-chip-V3 array [23]. A total of 59 cell lines, originating from breast, central nervous system, colon, leukemia, melanoma, Non-Small Cell Lung, ovarian, prostate, and renal tissues, were used in our study, ignoring the cell line “LC:NCI_H23” for lack of necessary meta-information. For a pre-filtration of non-informative molecules, we only analyzed ‘frequently expressed’ miRNAs and mRNAs whose log2-based expression values were larger than the dataset-specific minimum value (2.3 and 7 for miRNA and mRNA respectively) in more than 47 (~80%) arrays. Afterwards, values in each row (corresponding to each mRNA or miRNA) of the two expression datasets were centered to zero, and rows of synonymous probes were averaged to designate a unique miRNA or mRNA. Finally, we determined two parallel expression datasets across a same spectrum of cancer cell lines, one involving 195 microRNA genes and another involving 8388 protein-coding genes.

### **Candidate miRNA-to-gene regulatory relationships from miRGen**

From the miRGen website (http://www.diana.pcbi.upenn.edu/miRGen, version 3.0), we obtained 118,408 human candidate miRNA-gene regulatory relationships involving 276 human microRNAs and 10,255 targets, which were a union of results from three forward predicting algorithms: PicTar [30], TargetScan [5] and miRanda [31]. For more information, please refer to our related work [27] and its supplement 2.

### **Candidate TF-to-gene regulatory relationships from UCSC and TRED**

A set of forward predicted TF-gene regulatory relationships were compiled by merging records from UCSC (http://genome.ucsc.edu/) and TRED (http://rulai.cshl.edu/TRED/), which included 130,338 binary tuples involving 214 human TFs and 16,534 targets. For more information, please refer to our related work [27] and its supplement 3.

### **Candidate TF-to-miRNA regulatory relationships from UCSC**

The file ‘wgRna.txt’, downloaded from UCSC hg18, gave coordinate information of human microRNA genes. For all miRNAs, around 35% were embedded in protein-coding gene regions and on the same-strand of the host gene, i.e., intragenic, whereas the others were located outside protein-coding gene regions, i.e., intergenic. Note that miRNAs embedded in protein gene regions but on the opposite strand to the host gene’s were assigned to the so-called ‘intergenic’ group.

For intragenic miRNAs, we assume that they have the same transcriptional factors as their host genes considering the co-transcription of intragenic miRNAs and the hosts [32,33]. This is a common tactic in this field [28], though some violations were lately observed [34]. For intergenic miRNAs, we inherited pioneer operations [35] to group them into genomic clusters where in each cluster every two proximate miRNAs were separated by not more than 7.5 kb (more explanation is found in Additional file 1). Then we investigated the distribution of TF binding site (TFBS) near the first microRNA in each cluster and defined -3Kb to +1Kb of the first microRNA's transcription starting site (TSS) as the promoter region of the whole cluster (more explanation on the promoter definition is found in Additional file 1.) On these grounds, candidate TF-target microRNA relationships for intergenic miRNAs were established.

### **Network modeling**

The simplicity of the linear regression model has led many groups to employ it to reveal the gene network from gene expression data (for a review please see [10]), and it was also successfully utilized in our related work [27]. In this work, still by using the linear regression models, we constructed a combinatorial gene regulatory network based on the forward predicted regulation relationships involving TFs and miRNAs and 59 different expression values for each of 199 miRNAs and 8388 protein-coding genes. We noted that in the forward predictions, a target can be regulated by quite a lot of regulation factors: for instance, PLEC1, DMD, and BDNF were associated with more than 50 TFs, inconsistent with the observation that a gene is rarely regulated by more than 20 regulators [7]. As we did previously [27], we therefore conducted a single variable linear regression (Equation 1) with respect to every pair of a regulator (TF or miRNA) and a putative target, in order to filter out the regulation relationships where the regulator was not significantly related to the concerned target. For a specific target *t* (an mRNA or a miRNA), its expression level (log2 expression value), $E_{t}$, is modeled in Equation 1. $E_{r_{t}}$ and $A_{r_{t}}$ respectively stand for the observed expression level and the to-be-estimated regulatory efficacy of a regulator *r*_*t*_ (a miRNA or a TF) which regulates the target *t*. ‘*Intercept*’ is a constant, and *err*, following a normal distribution with a zero mean, captures the variation of gene *t*'s mRNA level that cannot be interpreted by its regulators.

(1) $$E_{t}=E_{r_{t}}\cdot A_{r_{t}}+\text{intercept}+\text{err}$$

After regression of Equation 1, TFs and miRNAs without statistically significant relationship (p > α_1_, *α*_*1*_ = 0.05) with their putative targets were disconnected with their putative targets. With regards to miRNA-to-target regulations, specially, we only kept the ones with negative regulation efficacy $A_{r_{t}}$ . In this way, the number of putative regulation relationships was largely cut down. The remaining regulators for a specific target, mRNA or miRNA, were represented as independent variables in the formal multivariate linear regression (Equation 2 or Equation 3).

The expression level (log2 expression value) of a mRNA target *g*, $E_{g}$ , is modeled in Equation 2. $A_{tf_{g}}$ is a vector of the regulating efficacy of TFs that regulate gene *g* (*tf*_*g*_); correspondingly, $E_{tf_{g}}$ is a vector of the mRNA levels (log2 expression value) of those TFs. Similarly, $A_{m_{g}}$ is a vector of the regulating efficacy of miRNAs that regulate gene *g*, and $E_{m_{g}}$ is a vector of the levels (log2 expression values) of those miRNAs. Note that miRNAs here were assumed to regulate their targets only through mRNA degradation, which is acceptable as mammalian miRNAs predominantly act to decrease target mRNA levels through mRNA degradation rather than mRNA blockage [36].

(2) $$E_{g}=E_{tf_{g}}\cdot A_{tf_{g}}+E_{m_{g}}\cdot A_{m_{g}}+\text{intercept}+\text{err}$$

Similarly, the expression level (log2 expression value) of a miRNA target *m*, $E_{m}$ , is modeled in Equation 3 with $E_{tf_{m}}$representing the expression levels of its regulating TFs, and $A_{tf_{m}}$ representing their regulation efficacies.

(3) $$E_{m}=E_{tf_{m}}\cdot A_{tf_{m}}+\text{intercept}+\text{err}$$

In brief, expression levels of mRNAs (Equation 2) and miRNAs (Equation 3) were modeled by the regulators that passed the statistical test (p < =*α*_*1*_) in the pre-filtering step (Equation 1), and the stepwise linear regression was implemented to determine the ultimate regulators of a particular target. As we did for Equation 1, if a positive miRNA-to-target regulation efficacy appears in the final regression model of Equation 2, all regulations reserved in the model for the same target gene *g* were eliminated.

An R function implementing this network modelling algorithm (*miRNAII.regression*) is provided in the supplementary source codes (Additional file 2).

## **Results**

### **A genome-scale combinatorial gene regulatory network in human cancer**

**Figure 1:**
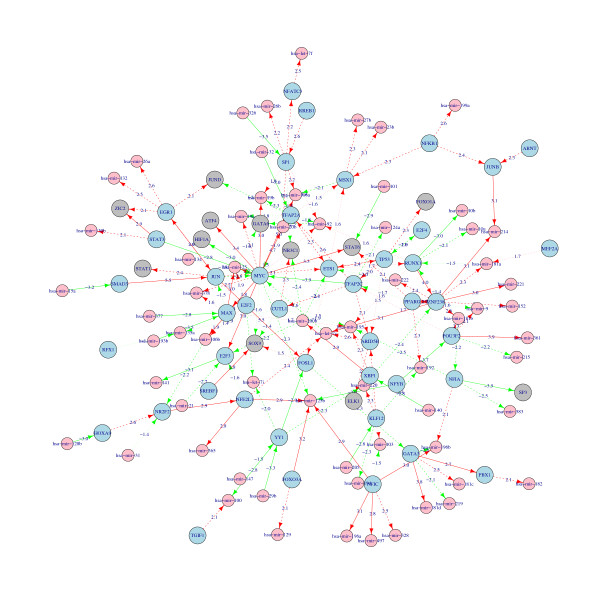
**A sub-network from the human cancer combinatorial gene regulatory network composed of regulators only.** Pink nodes indicate miRNAs; blue and grey nodes indicate TFs regulating or not regulating other regulators. Regulation relationships are differentially designated with three different edges according to the linear regression p-value: solid lines (most credible, p < 0.01), dashed line (medium credible, 0.01 < p < 0.05), and dotted line (less credible, p > 0.05). Numbers tagged with each edge are the T-statistics of the corresponding regulation.

By integrating the forward-predicted regulatory relationships and the miRNA/mRNA expression data with the linear regression models (Equations 1, 2, and 3), we culled a subset of regulatory relationships that were hopefully more plausible than the beginning set of forward-predicted regulatory relationships. These remaining regulator-target relationships make up a combinatorial network of transcriptional regulations and miRNA regulations in human cancer, which involves 3418 vertices and 5136 edges. The full information on the edges and vertices are included in Additional file 3: Table S1 and Additional file 4: Table S2, while the summary statistics are shown in Table 1. A subnetwork that is composed solely of regulation factors (TFs and miRNAs) is extracted from the whole network for a quick glimpse (Figure 1).

**Table 1:** Statistics of vertices and edges in the combinatorial gene regulatory network in human cancer

		**Number of unique objects**
Vertices	MiRNAs	159 (101 as only regulator, 5 as only target, and 53 as both)
	TFs	81 (22 as only regulator, 13 as only targets, and 46 as both)
	Non-TF protein-coding genes	3178
	Total	3418
Edges	miRNA-gene	1625
	TF-gene	3413
	TF-microRNA	98
	Total	5136

To estimate the false discovery rate of our predicted regulatory relationships, we randomly permuted the expression values within each expression dataset and conducted the linear regression procedure (Equations 1, 2, and 3) over the randomized miRNA/mRNA expression datasets for 100 times. The regulation edges in the combinatorial networks resulting from the randomized datasets were deemed as false discoveries. In this way, we estimated that the overall false discovery rate (FDR) of the regulation relationships remaining in the human cancer combinatorial gene regulatory network were 17.2% (Table 2).

**Table 2:** False discovery rates (FDRs) of predicted regulation relationships

**Regulation type**	**Edges in shuffled network**	**Edges in predicted network**	**FDR (%)**
miRNA → gene	477.8(+ − 29.2)	1625	29.4(+ − 1.8)
TF → gene	382.3(+ − 23.9)	3413	11.2(+ − 0.7)
TF → miRNA	21(+ − 5.2)	98	21.5(+ − 5.3)
Overall	881.1(+ − 46.2)	5136	17.2(+ − 0.9)

We also evaluated our methods in terms of the predictability of the resultant linear model (Equation 2 and Equation 3). While in real work we made use of all 59 expression data-points of a gene, in the jack-knife like evaluation procedure we excluded one data-point from the modeling (Equations 1, 2, and 3) and used the fitted model (Equation 2/Equation 3) to predict the left-out data-point. For each target gene we had 59 iterations, and we calculated the Pearson correlation coefficient (PCC) between the measured expression values and the predicted ones. As a result, significantly higher PCCs were obtained with real expression data than with randomly permuted data (Additional file 5), indicating that the obtained linear model, or roughly speaking the combination of included regulations, had a significant power to predict the targets’ expression values.

Finally, by resorting to databases TarBase, TransFAC, and TransmiR which contain different types of experimentally validated regulation relationships, we compared the fractions of experimentally supported records in the modeling results and in the forward predictions. It turned out that the fractions had been significantly raised by our integrative modeling strategy for all three regulation types (Table 3 and Additional file 3: Table S1), proving the validity of our model.

**Table 3:** Validation of regulation relationships

**Regulation type**	**Validation source**	**Engineering method**	**Total items**	**Items validated**	**Validation fraction**	**Significance of validation fraction increase (binomial test p-value)**
miRNA-to-gene	TarBase	Forward	86,196	583	0.6%	<0.055
		Forward and reverse	1,625	16	1%	
TF-to-gene	TransFAC	Forward	58,417	137	0.3%	3.3e-13
		Forward and reverse	3,413	35	1%	
TF-to-miRNA	TransmiR	Forward	1195	23	1.9%	1.8e-5
		Forward and reverse	98	9	9.1%	

*Forward: the forward-predicted regulatory relationships involving only the regulators showing up in the combinatorial regulatory network; forward and reverse: the regulatory relationships showing up in the combinatorial regulatory network.

### **Key players in the human cancer combinatorial gene regulatory network**

Since our modeling work was based on the NCI60 expression data panel, the genes in the combinatorial regulatory network should be substantially related to cancer. For a total of 427 cancer related genes downloaded from the “Cancer Gene Census" (http://www.sanger.ac.uk/genetics/CGP/Census/), 197 overlapped the 8,388 frequently expressed genes of the NCI-60 mRNA expression dataset, of which 121 were found in the 3,259 genes remaining in the resultant regulatory network (Additional file 3: Table S1). The fraction of cancer-related genes was significantly raised from 2.3% to 3.7% (hypergeometric t-test p < e-10), consistent with the expectation that our gene regulatory network should enrich genes related to cancer. In the following sections we pinpointed the most noteworthy cancer-related genes and miRNAs from the topological viewpoint (for a full table of vertex properties see Additional file 4: Table S2).

### ***MYC***

From the combinatorial regulatory network, we concentrated on the most common regulators according to vertices’ out-degrees and betweennesses [37] (Additional file 4: Table S2). Among these common ones, MYC has the largest number of regulating targets (301) and the highest betweenness (46215.9). Besides, of all regulators, MYC has the largest in-degree (six), implying that MYC is under strict control. These statistics are consistent with the unique role of MYC in tumorigenesis. As one of the most important cancer-related genes, MYC has been proved to participate in several essential functions, such as cell cycle progression and apoptosis. Particularly, MYC was found to be involved in the regulation of a broad range of miRNAs, many of which play key roles in cell proliferation and oncogenic transformation [38,39].

In our network, MYC was predicted to regulate 10 miRNAs: miR-378, hsa-miR-17, hsa-miR-19a, hsa-miR-19b, hsa-miR-20b, hsa-miR-92, hsa-miR-106a, hsa-miR-25, and hsa-miR-106b, and hsa-miR-125b. Of them, hsa-miR-378 is located in the intron of protein-coding genes PPARGC1B, an experimentally validated transcriptional targets of MYC [40], and another eight miRNAs (hsa-miR-17, hsa-miR-19a, hsa-miR-19b, hsa-miR-20b, hsa-miR-92, hsa-miR-106a, hsa-miR-25, and hsa-miR-106b) belong to three paralogous clusters located on chromosome 13 (the hsa-miR-17 cluster), chromosome X (the hsa-miR-106a cluster), and chromosome 7 (the hsa-miR-106b cluster), with the former two clusters having been proved to be regulated by MYC [41]. Finally, our prediction of hsa-miR-125b being repressed by MYC was in accordance with an independent observation [42]. It is notable that, except hsa-miR-125b, the other nine miRNAs were all predicted to be ‘ACTIVATED’ by MYC, and this seems contradictory to a previous notion that ‘widespread microRNA REPRESSION by Myc contributes to tumorigenesis’ [42].

Aside from the 10 miRNA targets, MYC demonstrated another 291 protein-coding genes as its regulating targets. In order to evaluate the reliability of our refined MYC targets, we referred to a set of 3,455 c-Myc binding targets determined in human B lymphoid tumor using chromatin immunoprecipitation coupled with pair-end ditag sequencing analysis (ChIP–PET) [40], and found that the fraction of ChIP-PET confirmed MYC targets was increased from 18.0% (453 of 2508) in the forward prediction to 23.0% (67 of 291) in the modeling result, which was a statistical significant enrichment (hypergeometric test p = 0.009). The 67 MYC targets confirmed by ChIP-PET experiment were marked out in Additional file 3: Table S1.

#### ***Hsa-miR-106b and hsa-let-7c***

By analogy to MYC, miR-106b, a target of MYC, is probably the most important miRNA since it has the largest number of targets among all miRNAs in the network. For the 44 predicted targets of miR-106b, 38 were covered in the public dataset GSE6838 (http://www.ncbi.nlm.nih.gov/projects/geo/query/acc.cgi?acc=GSE6838) recording the gene expression changes in cells transfected with hsa-miR-106b, and 21 were among the top 5% down-regulated genes (Additional file 6). While the hsa-miR-106 family have been implicated in breast cancer [43] and gastrointestinal tumor [44], our results furthermore suggest it may be a central miRNA underpinning the general tumorigenesis mechanism. In-depth investigations of hsa-miR-106b and its regulations are necessary in future studies of fundamental cancer mechanisms.

In our network, there are some miRNAs that have betweenness comparable to TFs (Additional file 4: Table S2), among which hsa-let-7c is a typical example. The betweenness of hsa-let-7c is ranked after only four TFs in the combinatorial regulatory network, and its confirmed inhibition of MYC [45] happened to have the highest edge-wise betweenness. In addition, hsa-let-7c is regulated by NFE2L1, the 5th TF with the largest out-degree. All these observations indicate that hsa-let-7c is another important miRNA probably underpinning the general tumorigenesis mechanism.

### **Global and local architecture of the human cancer combinatorial regulatory network**

#### ***A hierarchical scale-free network***

**Figure 2:**
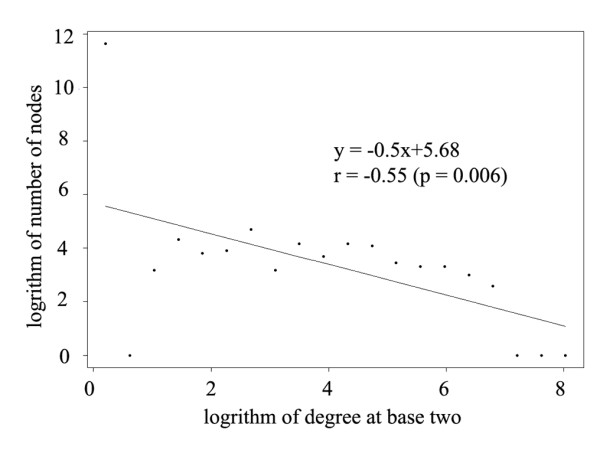
Log-scale distribution of out-degrees of the human cancer combinatorial gene regulatory network.

In the human cancer combinatorial gene regulatory network, the numbers of inward and outward regulations made the in-degree and out-degree of a vertex respectively. Firstly, it was found that while a regulator could regulate more than one hundred targets, a gene was regulated by at the most six regulators. The in-degrees and out-degrees in our network were much lower than previous reports [7,8], partly because previous regulatory networks were constructed as ‘reference networks’ that spanned across all spatiotemporal contexts. Secondly, a larger in-degree was found to be associated with a larger out-degree (Pearson correlation coefficient 0.4306, p value = 3.297e-11), suggesting that a regulator regulating more targets is subjected to regulations from more regulators. Thirdly, out-degrees of all vertices seemed to form a power-law distribution with a slope of −0.5 (p = 0.006) (Figure 2). This is accordant with our expectation, as similar observations have been made in the regulatory networks of protein-coding genes or miRNAs separately [46,47]. Finally, with a Krackhardt Hierarchy Score [48] of 0.998, the combinatorial gene regulatory network turned out to be a hierarchical scale-free network, consistent with previous studies of metabolic networks [49], protein-protein interaction networks [50,51], and transcriptional regulation networks [52].

#### ***Coordinating TF-TF, miRNA-miRNA, and TF-miRNA pairs***

It is of great interest how regulators coordinate to regulate their targets. In our combinatorial regulatory network, we identified coordinating regulator pairs which share a significantly large number of common targets (one-sided Fisher’s exact test, p < 0.01), and obtained 17 TF-TF pairs, 46 miR-miR pairs and 17 TF-miR pairs (Additional file 7: Table S3). To our expectation, coordinated regulations often take place among regulators who usually form complexes and play their roles as a whole, such as MYC and MAX; NFKB1 and RELA; JUN, JUND, and FOSL1. Regulators from a same protein family or miRNA family are also likely to form coordinating regulators, such as those from the E2F family, the STAT family, or the let-7 family.

Our 21 miR-miR co-regulating pairs were compared with the counterpart set of 199 pairs reported earlier in a forward-prediction work [7]. There was not a single pair that perfectly matched between the two sets, and, if we relaxed the matching criteria to the family level, only three of our 46 pairs (hsa-mir-130a and hsa-mir-152; hsa-mir-130a and hsa-148b; has-mir-130b and has-mir-19a) showed up in the previous set of 199 pairs. It seems that important discoveries may differ between a forward-predicted reference network and a reverse-engineered context-specific network, which warrants the efforts to integrate the forward-predicted putative regulation relationships with various conditional expression datasets so as to construct conditional combinatorial gene regulatory networks that are specific to different experimental conditions.

#### ***Recurrent feed-forward loops and feed-backward-loops***

By definition, there are 18 types of closed triple-vertex regulatory circuits that involve at least a miR and a TF, which can be classified into ‘feed-forward loops’ (FFLs) and ‘feed-backward-loops’ (FBLs) by considering the connecting ways of directed regulations [28]. Previous studies found that TF-initiated or miRNA-initiated feed-forward loops (FFLs) may be characteristic regulatory motifs in tumor cells influencing a large number of target genes from specific biological pathways [53,54]. Therefore we counted in our cancer regulatory network the occurrences of all possible fourteen triple-vertex FFLs and four FBLs, and estimated the corresponding p-values through counting the counterpart occurrences 1000 times in randomly shuffled networks. The randomization was achieved by randomly shuffling the actual regulatory relationships (TF-miRNA, TF-mRNA, and miRNA-mRNA), provided that the type-specific in-degree and type-specific out-degree of each vertex were fixed, with the type being miRNA or mRNA.

**Figure 3:**
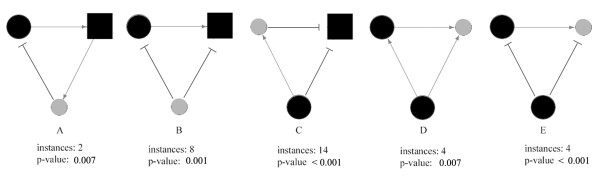
**Significantly recurrent feed-backward-loop (A) and feed-forward loops (B, C, D, and E).** Light-grey circle: miRNA; dark-grey circle: transcription factor (TF); dark-grey rectangle: non-TF protein-coding gene. Arrow-headed line: activating regulation; hammer-headed line: repressing regulation.

Of the 18 motifs being surveyed, one FBL and four FFLs turned out to be significantly recurrent (p < 0.01, Figure 3). Composed of one miR-TF post-transcriptional regulation and two positive transcriptional regulations, the only one significant FBL (Figure 3A) is a negative feedback loop potentially able to maintain stable volume of key molecules [54]. For the four significant FFLs, one has a miRNA as the foremost regulator (Figure 3B), another has a miRNA as an interim transmitter of regulation signal (Figure 3C), and the other two have miRNA as the ultimate target (Figure 3D and E). The last two FFLs differ from each other in the overall regulation effect: positive or negative. In each significant FFL, the multiple regulation cascades are always coherent to each other (Figure 3B, C, D, and E). At last, we noticed that most instances of these significant FFLs include the gene MYC. As a matter of fact, the issue of MYC-involved regulatory circuits was specially addressed and a curated database of MYC-involved and miRNA mediated FFLs was released to public very recently [55].

## **Discussion**

In this work, we made use of parallel miRNA and mRNA expression datasets from the NCI-60 data panel, and, by using the linear regression model as a tool to integrate the sequence-matching information and the expression data, we sifted the forward-predicted regulation relationships and constructed a human cancer gene regulatory network composed of transcriptional control and miRNA control. This differs from related peer works mostly in that we realized for the first time a global landscape of combinatorial gene regulatory network in a specific biological context. By analyzing the results originating from randomized expression datasets, we estimated that the false discovery rate of our selected regulations were 17.2%, and by taking the experimental evidences from TransFAC, TarBase, and TransmiR as benchmarks, we proved that our human cancer combinatorial gene regulatory network tended to enrich genuine regulations of all three types.

Two years ago, we integrated the forward predictions with the miRNA-perturbed gene expression datasets (MPGE datasets) to elucidate the miRNA-centered primary and secondary regulatory cascades in human cancer, which encompassed two types of mRNA regulation, miRNA-to-mRNA and TF-to-mRNA [27]. By introducing parallel miRNA and mRNA expression datasets, here we manage to map a combinatorial gene regulatory network that encompass one more regulation relationship: the regulation of miRNA by TF, and as a result, TF control and miRNA control are comprehensively described in this genome-scale cancer-related network. With the microarrays becoming cheaper and next-generation sequencing platforms being rapidly developed, it is foreseeable that a large amount of parallel miRNA and mRNA expression datasets are attainable in the near future, and thus, our modeling strategy can be extended to enormous cell types, strains, tissues, and so on.

In human cancer, miRNAs are presumed to preferentially couple its post-transcriptional inhibition with TF-initiated transcription in combinatorial regulatory circuits [53,54]. Our regulatory network provides a holistic background in which the important elements, relationships, and network motifs can be analyzed thoroughly. For instance, a quick topology analysis of this network highlights the very important cancer-related transcription factor MYC and two remarkable miRNAs hsa-miR-106b and hsa-let-7c. While these entities themselves have already been found to be cancer relevant, our network demonstrates their putative regulatory contexts comprising their coordinating partners and their targets, and such information may shed additional light on tumorigenesis mechanisms. For instance, we discriminated three significantly recurrent coherent FFL motifs from our combinatorial regulatory network, most of which involve the ascertained cancer-related transcription factor MYC. Coherent FFLs are often used to amplify target genes or reduce target genes to inconsequential levels. These types of regulation are often being used as an "on/off" switch during developmental transitions and cellular differentiation. Current work in model organisms suggested miRNAs and TFs are also involved in incoherent FFLs - miRNAs appear to buffer against biological noise by targeting several components within a network in an incoherent manner [54]. The fact that incoherent FFL is missing in our cancer regulation network may imply an important and specific aspect of cancer cells.

While the overall working pipeline turns out to be effective in this work, some specific steps need to be discussed. For example, the prefiltration step (Equation 1) is incurred primarily for reducing computation complexity of the following steps (Equation 2/Equation 3) or circumventing the n < p problem (if the number of samples is less than the number of putative regulators, the stepwise linear regression is unsolvable). Taking into account the forthcoming reduction in microarray or RNA-seq costs, the number of samples in future parallel expression datasets may be larger, which may greatly alleviate the pre-filtration pressure. For another example, doubts have been arising with regards to the stepwise (STEP) linear regression [56], and therefore it is necessary to consider other variants for modeling Equation 2, say the ‘least absolute shrinkage and selection operator (LASSO)’[57]. LASSO is a penalized linear regression model which shrinks the coefficients of some predictors to smaller values or zeroes, and therefore can be used as a variable selection tool. On the same input data materials, we implemented the algorithm using STEP and LASSO separately, and got two different combinatorial regulation networks. We found that overall LASSO gave rise to a network with denser edges (three edges per node for LASSO, in comparison to two edges per node for STEP). The estimated false discovery rates were 23.3% for LASSO, a little bit higher than that of STEP. When comparing the LASSO-based results and the step-based results, we found on the whole a high level of mutual consistency (data not shown). It should be noted that this comparison was limited to a particular sets of datasets relating to the NCI60 case, and more rigorous comparative experiments are needed to substantiate an optimal choice of the regression model in this step. In the supplementary source codes, we allow the user to decide whether to implement the prefiltration Equation 1 or not, and provide both the STEP portal and the LASSO portal for Equation 2.

Towards the ultimate goal of ‘differential networking’ analysis [58], we made the first step to map a TF and miRNA-involved combinatorial gene regulation network for a specific context, human cancer. Confined to the used expression dataset, we arrived at results for the general tumorigenesis but not for a specific cancer type; and the results reported herein would be consolidated if a direct comparison was made between the cancer-specific network and a ‘normal’ network. Given the ever-increasing resource of parallel miRNA and mRNA expression datasets enabled by rapidly developing RNA-seqs, improvements are expected to be made in forthcoming succeeding works.

## **Conclusions**

In this work, we made an attempt to integrate the forward engineering and reverse engineering strategies and for the first time resulted in a global landscape of combinatorial gene regulatory network in a specific biological context (human cancer) that has a moderate false discovery rate and is enriched with confirmed regulations. The human cancer combinatory gene regulatory network is found to be a hierarchical scale-free network with MYC, hsa-miR-106b and has-let-7c being the most important regulators. From the network, 17 TF-TF pairs, 46 miR-miR pairs and 17 TF-miR pairs are identified as the significantly co-regulating regulator pairs, and four triple-vertex regulatory circuits (one FBL and three FFLs) turn out as significantly recurrent building motifs. We believe our work provides a scaffold combinatorial gene regulatory network allowing systematic study of cancer gene regulation, and that our pipeline can be extended to reveal conditional combinatorial regulatory landscapes correlating to specific cellular contexts.

## **Competing interests**

The authors’ declare that they have no competing interests.

## **Authors’ contributions**

HY and KT contributed to the design and conception of the study, conducted computational experiments, analyzed and interpreted data and drafted the manuscript. YJW and JZM joined in the processing of data materials and wrote part of the computer codes. LX, YYL and YXL conceived of the project and participated in its design, helped to analyze and interpret the data and drafted the manuscript. All authors have read and approved the manuscript for publication.

## Supplementary Material

Additional file 1 How we compiled TF-to-miRNA regulation relationships for intergenicmiRNAs. Click here for file

Additional file 2 The source codes/R functions for this study. Click here for file

Additional file 3 **Table S1**The edges of the human cancer combinatorial gene regulation network. Click here for file

Additional file 4 **Table S2**The vertices of the human cancer combinatorial gene regulation network. Click here for file

Additional file 5 The evaluation of predictability of our linear regression models. Click here for file

Additional file 6 Validation of part of predicted hsa-miR-106b targets by a miRNA-transfection dataset GSE6838. Click here for file

Additional file 7 **Table S3.** Significantly coordinating regulator pairs from the human cancer combinatorial regulatory network. Click here for file
