# Supplementary material for: Combinatorial network of transcriptional regulation and microRNA regulation in human cancer
Source: BMC Syst Biol. 2012 Jun 12;6:61. doi: 10.1186/1752-0509-6-61 (PMC3483236; doi:10.1186/1752-0509-6-61)
Supplement: Additional file 1 — How we compiled TF-to-miRNA regulation relationships for intergenicmiRNAs. [file 1752-0509-6-61-S1.doc]

**How we compiled TF-to-miRNA regulation relationships for intergenic miRNAs**

Additional File to “human cancer combinatorial gene regulatory network”

For intergenic miRNAs, we first organized them into clusters where in each cluster every two adjacent miRNAs on the same chromosome are not separated by more than 7.5 kb. The inter-miRNA distance threshold, 7.5k, was determined according to the following Figure 1.

Given a miRNA cluster, the 5’- starting position of the most upstream miRNA (the first one on the ‘+’ strand; the last one on the ‘-’ strand) was deemed as the TSS of the whole miRNA cluster, and the [-3K, 1K] region was defined as the promoter of the miRNA cluster. TFBSs falling in this region were associated with all members of the cluster, and so do the TFs binding to these TFBSs.


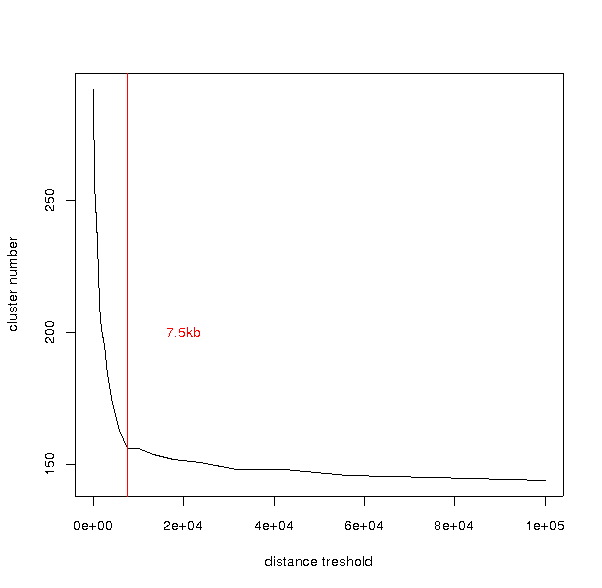


Figure 1. The dependence of miRNA cluster number on the inter-miRNA distance threshold. On a linear chromosome, two miRNAs were assigned to one cluster if the distance between them was less than inter-miRNA distance threshold (the x axis). Raising x would lower the number of the resulted clusters (the y axis), as reflected by the black curve. The y’s decreasing velocity slowed down significantly after about x=7.5kb.


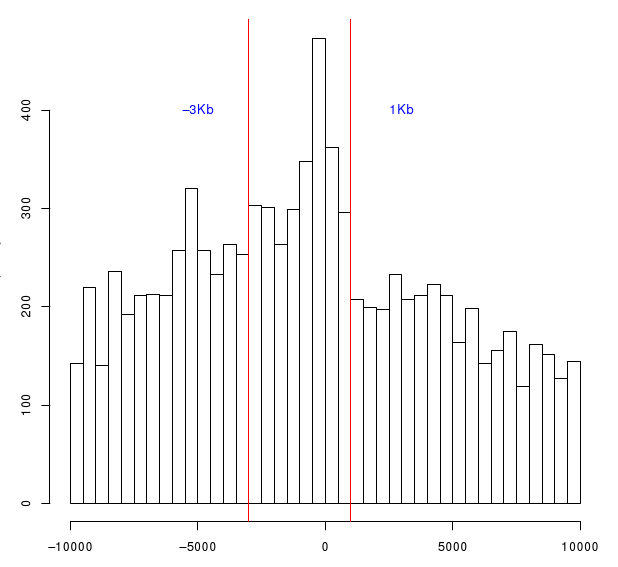


Figure 2. TFBS distribution for inter-genetic microRNA clusters

Distribution of TFBS with +-10kb near inter-genetic microRNA clusters’s putative TSS. The X axis indicates the relative distance to a miRNA cluster’s putative TSS. The negative digit indicates upstream while the positive indicates downstream. The Y axis indicates how many TFBSs (the frequency) fall into each segment of the [-10k, 10k] range as distributed on the X axis. The two red vertical lines delimit the [-3k, 1k] core region that has the highest TFBS density.
