# Supplementary material for: Combinatorial network of transcriptional regulation and microRNA regulation in human cancer
Source: BMC Syst Biol. 2012 Jun 12;6:61. doi: 10.1186/1752-0509-6-61 (PMC3483236; doi:10.1186/1752-0509-6-61)
Supplement: Additional file 5 — The evaluation of predictability of our linear regression models. [file 1752-0509-6-61-S5.docx]

evaluation of predictability of our linear regression models

Additional File to “human cancer combinatorial gene regulatory network”

We also evaluated our methods in terms of the predictability of the resultant linear model (Eq. 2 and Eq. 3). While in real work we made use of all 59 expression data-points of a gene, in the jack-knife like evaluation procedure we excluded one data-point from the modeling (Eq. 1, 2, and 3) and used the fitted model (Eq. 3) to predict the left-out data-point. For each target gene we had 59 iterations, and we calculated the Pearson correlation coefficient (PCC) between the measured expression values and the predicted ones. As a result, significantly higher PCCs were obtained with real expression data than with randomly permuted data (the following figure), indicating that the resultant linear models successfully represented the regulation relationships underlying integrated expression information of mRNA and miRNA.


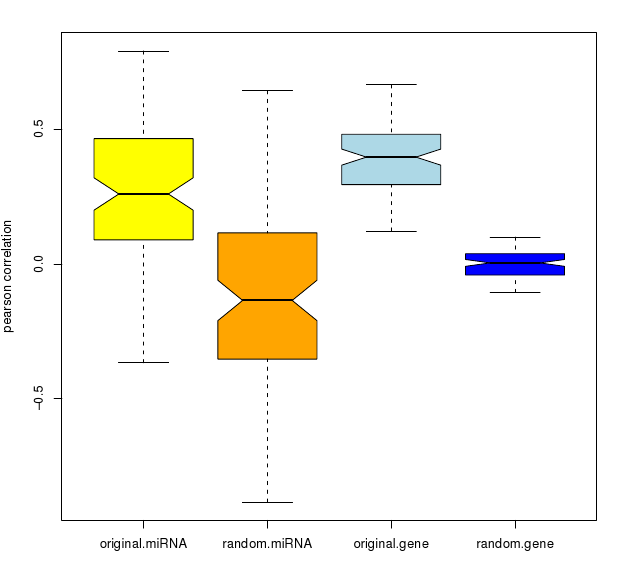


Figure. Pearson correlation coefficients (PCCs) between the predicted expression values and the measured values are significantly higher for models based on real expression datasets (1^st^ and 3^rd^ boxplots) than models on randomized datasets (2^nd^ and 4^th^ boxplots).
