# Supplementary material for: Combinatorial network of transcriptional regulation and microRNA regulation in human cancer
Source: BMC Syst Biol. 2012 Jun 12;6:61. doi: 10.1186/1752-0509-6-61 (PMC3483236; doi:10.1186/1752-0509-6-61)
Supplement: Additional file 6 — Validation of part of predicted hsa-miR-106b targets by a miRNA-transfection dataset GSE6838. [file 1752-0509-6-61-S6.doc]

**Validation of part of predicted hsa-miR-106b targets by a miRNA-transfection dataset GSE6838**

Additional File to “human cancer combinatorial gene regulatory network”

For the 44 predicted targets of miR-106b, 38 were covered in the public dataset GSE6838 (http://www.ncbi.nlm.nih.gov/projects/geo/query/acc.cgi?acc=GSE6838) recording the gene expression changes in cells transfected with mir-106b, and 21 were among the top 5% down-regulated genes (see the following figures A and B).


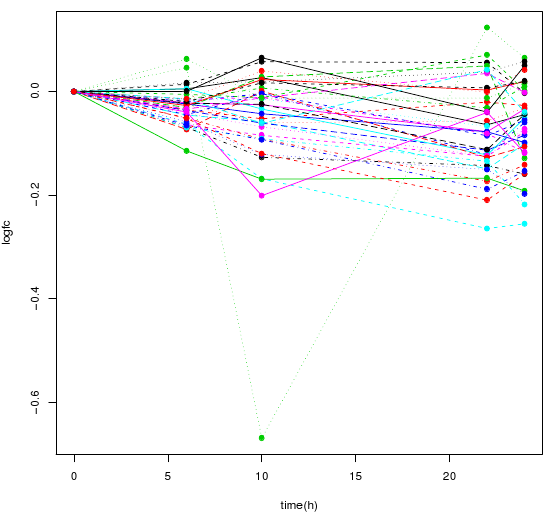


1. mRNA level changes of 38 predicted target genes of has-mir-106b at four different time points after transfection of it into # cell lines. Each curve corresponds to the expression time expression series of a gene.


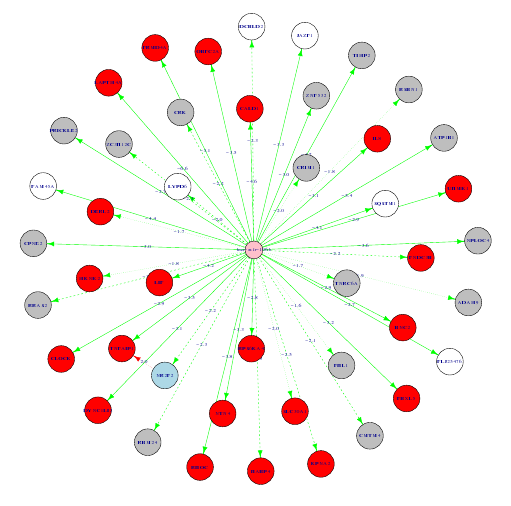


B) The sub-network of regulations initiated from hsa-mir-106b, extracted from the human cancer combinatorial gene regulation network. The red vertexes were predicted targets that were validated in the GSE6838 transfection experiment (expression changes at one or more time points are among the top 5% down-regulated values). The gray vertexes were predicted targets not validated in the transfection experiment and the white vertexes were predicted targets not involved in the transfection experiment.
